# Primary healthcare delivery models in African conflict-affected settings: a systematic review

**DOI:** 10.1186/s13031-023-00533-w

**Published:** 2023-07-15

**Authors:** Lundi-Anne Omam, Elizabeth Jarman, Kelli N. O’Laughlin, Rosalind Parkes-Ratanshi

**Affiliations:** 1 Department of Public Health and Primary Care, University of Cambridge, Cambridgeshire, UK; 2 Department of Psychiatry, University of Cambridge, Cambridgeshire, UK; 3 Health Department, Reach Out Cameroon, Buea, Cameroon; 4 Infectious Diseases Institute, Makerere University College of Health Sciences, Kampala, Uganda; 5 Departments of Emergency Medicine and Global Health, University of Washington, Seattle, USA

**Keywords:** Primary healthcare, Conflict-affected, Models of care, Africa

## Abstract

**Background:**

In conflict-affected settings, access to primary healthcare for displaced populations is constrained by multiple challenges. These include geographical, cultural, communication, logistical and financial barriers, as well as risks posed to health workers and the population by insecurity. Different models of care are used to provide primary healthcare to affected communities. However, there is a paucity of evidence on how these models are selected and implemented by organisations working in conflict and displacement-affected settings. Our aim was to explore the different primary healthcare delivery models used in conflict-affected settings to understand gaps in existing healthcare delivery models.

**Methods:**

We conducted a systematic review using the Preferred Reporting Items for Systematic Reviews and Meta-analyses guidelines. The review protocol was registered with the International Prospective Register of Systematic Reviews. We searched six databases for manuscripts published from January 1992 to December 2020. Publications were included if they reported primary healthcare models of care in conflict-affected settings of Africa. Data was analyzed descriptively and thematically using tables, charts and text.

**Results:**

Forty-eight primary research articles were included for analysis from which thirty-three were rated as “high” quality. The results showed that the models of care in place in these conflict-affected settings include health facility-based, community-based, mobile clinics, outreach and home visits. Primary healthcare for internally displaced persons and refugees is provided by a wide range of actors including national and international organisations. A range of services is offered, most commonly nutrition, mental health and sexual/reproductive health. Some organisations offer vertical (stand-alone) services, while others use an integrated service delivery model. Multiple cadres of healthcare workers provide services, frequently lay healthcare workers such as Community Health Workers.

**Conclusion:**

Understanding the different modalities of primary healthcare delivery in conflict-affected settings is important to identify existing practices and gaps in service delivery. Service delivery using community health workers in conflict-affected settings is a low-cost primary care delivery strategy that may help optimize contributions of existing personnel through task shifting.

**Supplementary Information:**

The online version contains supplementary material available at 10.1186/s13031-023-00533-w.

## Background

Primary healthcare is a whole-of-society approach to health and wellbeing aimed at attaining the highest possible level of health, centred on the preferences and needs of individuals, families and communities. Primary healthcare as defined by the World Health Organisation encompasses the entire continuum of health services from health promotion, disease prevention and treatment, rehabilitation and palliative care and should be delivered as close as feasible to the daily environment of people. Primary healthcare addresses the wider determinants of health while focusing on the comprehensive and interconnected aspects of social, mental, physical health and wellbeing [1]. In 2018, the Declaration of Astana at the Global Conference on Primary Healthcare marked a renewed political commitment from policy makers and the international community in achieving “health-for-all”[2]. This renewed vision also highlighted the important role of a primary healthcare approach for people living in conflict-affected settings [3].

Globally, over one billion people live in a fragile, violent or conflict-affected setting [4]. Conflict-affected settings are settings with political instability and armed conflict that are man-made and could either be acute, protracted or post-conflict. Acute conflict-affected settings are those with armed conflict below five years. Protracted conflicts are those with armed conflict exceeding five years. Post conflicts are those in which there is no active conflict and are undergoing periods of recovery [4, 5]. In 2017, it was estimated that 80,000 people worldwide were forced to flee their homes every day because of violence, conflict or disaster [6]. Most suffering in conflict-affected settings or settings affected by natural disasters, is due to population displacement, as people forcefully flee their homes [5]. Populations can either be displaced within their country (internally displaced persons; IDPs) or move across country borders (refugees) [5]. Regardless of the cause of displacement, displaced persons generally have limited or no access to healthcare yet bear considerable disease burden which negatively impacts their health needs [5–7]. Recent estimates in conflict-affected settings show that 53% of deaths in children under 5 years, 45% of neonatal deaths and 60% of preventable maternal deaths occur in fragile, violent or conflict settings [8]. Previous studies have shown poor quality of primary healthcare services as a result of disruption to services in conflict-affected settings [9].

In conflict-affected settings, the provision of healthcare by humanitarian organisations is guided by international law which mandates that accessible healthcare be provided to all without discrimination [10]. Further guidance is provided by the Sphere handbook which details the humanitarian charter and minimum standards in humanitarian response, and which also covers recommendations on healthcare provision in conflict-affected settings [11]. Often, providing healthcare in conflict-affected settings is difficult due to high population density, poor sanitation and hygiene services, inadequate access to potable water, and limited access to preventative and curative health services. Damaged infrastructure, physical and psychological trauma, and challenging living and economic conditions pose additional health risk for communities in conflict-affected settings. These challenges make refugees and IDPs particularly susceptible to infectious disease outbreaks [12, 13]. The difficulties faced by populations in conflict-affected settings to routinely accessing healthcare might be mitigated by provision of integrated primary healthcare services. Through integrated health services, holistic and comprehensive services can be provided to populations in conflict-affected settings using models of care which might be more accessible, safer, efficient, cost effective and high quality. The first and frontline healthcare responders in conflict-affected settings, are likely to be primary healthcare workers who provide care for infectious diseases, injuries, and other illness [3]. The care being provided is usually either vertical or integrated and includes the management of acute respiratory diseases, diarrheal diseases, HIV, malaria, acute malnutrition, measles, meningococcal diseases, tuberculosis, mental health, injuries and trauma and non-communicable diseases [5, 14, 15].

Globally, there are thousands of humanitarian organisations that provide healthcare interventions in conflict-affected settings [16]. When the ability of the government health ministries or agencies/departments to perform their functions are weakened or when the government loses overall capacity to carry out its functions due to armed conflict, humanitarian organisations often support the government to deliver health services in parallel or in some cases in total substitution using different models of care [9, 17, 18]. Examples of models of care used to provide primary healthcare in conflict-affected settings include health facility-based [17–20], community-based [21–23], outreach [17, 24, 25], mobile clinics [17, 19, 24] and home visits [26] (Table 1).

**Table 1:** Models of care and their definitions

Model of care	Definition
Health facility-based	A modality of delivering services offered by health personnel in fixed infrastructures like clinics, health centres and hospitals
Community-based	An approach that uses community health workers to deliver health services. Community health workers is an umbrella term used to refer to community members who are selected by their communities, receive low levels of formal education to provide healthcare in the communities where they live
Outreach	This a modality of service delivery referring to services delivered as an advance strategy of health service from an existing fixed health facility or away from the location where they usually work. This could either be provided by health facilities, mobile clinics, community health workers, private companies, non-governmental organisations, and Ministry of Health
Mobile clinics	Ambulatory approaches of providing preventive and curative health services on an intermittent base, operated by health personnel
Home visit	This is a modality of healthcare delivery in which the services are provided in the homes of the patients. Although home visits are carried out in community settings, it is a distinct modality of its own as it can be provided by both skilled and non-skilled health workers

As highlighted in Table 1 above, there are multiple overlapping models of healthcare provision in conflict-affected settings. We hypothesize that optimizing primary healthcare delivery in conflict-affected communities may be facilitated by careful selection of care delivery models in each setting. Towards exploring this hypothesis, we sought to review the literature describing models of care used by humanitarian organisations and governments (ministries of health) in conflict-affected settings in Africa. Our goal was to understand and document the different primary healthcare models used in conflict-affected settings. Factors considered included target populations, services offered, cost of services, human resources utilization, quality of services provided, accessibility of services, community engagement and sustainability. We limited our review to Africa, as the continent has the lowest health expenditure globally, and therefore the health system is more vulnerable to the effects of conflict. Additionally, Africa suffers from a low life expectancy driven by more infectious diseases such as HIV, malaria. These factors give rise to specific challenges in delivering healthcare in conflict-affected settings in Africa [27–29].

## Methods

### Protocol and registration

A systematic review was conducted in line with the standards of the Preferred Reporting Items for Systematic Reviews and Meta-Analyses (PRISMA) 2020 guidelines [30, 31]. The protocol for this review was registered with the International Prospective Register of Systematic Reviews (PROSPERO registration number: CRD42020224331).

### Literature search

From the 15th–16th December 2020, searches were conducted on Embase, MEDLINE, PubMed, Global Health, Web of Science, and Scopus using search terms related to primary healthcare delivery models in conflict-affected settings in Africa (See Additional file 1: Appendix 1 for full search syntax). Boolean logic operators “OR” and “AND”, together with truncations for key terms were used in these databases. Pre-identified key words and Medical Subject Headings (MeSH) terms were applied during the search across the databases. Articles and publications that were included in this study were those published from January 1992 to December 2020, as 1991 ties with the year the Inter-Agency Standing Committee (IASC) was established by United Nations (UN) and non-UN humanitarian partners for the coordination of humanitarian assistance[32].

### Inclusion and exclusion criteria

Studies that were included in this review were primary quantitative and qualitative studies on primary healthcare services with models of care. This included observational studies, randomised controlled trials, non-randomised controlled trials, and economic studies. Other peer-reviewed published studies were also included in the review. Opinion pieces, systematic reviews or narrative review articles were excluded from this review. More so, publications with the following outcomes were included: description of models of care, target population, services offered, cost, benefit package, involved, human resources, sustainability of model of care and challenges of model of care. Articles not in English, or reported on studies conducted outside Africa, or in Africa but not in conflict-affected settings, were not included in this review. Conference abstracts or studies published before 1992 were not included.

### Data extraction and synthesis

Article review, selection and extraction of data was done by two authors (LAO and EJ). After removing duplicate articles on Endnote (Clarivate, Philadelphia), the authors screened titles and abstracts independently using Rayyan (Rayyan.ai) to ensure their content were related to the inclusion criteria of the study. Next, a full review of each relevant manuscript was conducted independently, yielding the final selection of articles for this study. Discrepancies that arose during the selection of articles was resolved by the third and fourth authors (KO and RP). A tailored data extraction form was designed on an excel spreadsheet to collate data on the type of model of care, implementing institutions, population targeted, phase of conflict, services offered, benefit package, human resources involved. We did not restrict papers to original research papers but included other peer-reviewed published studies describing health interventions led by humanitarian organisations to gain more insights on the primary healthcare models used in conflict-affected settings in Africa.

### Quality assessment

The quality assessment of the selected articles was conducted using Critical Appraisal Skills Program (CASP) [11] and the Critical Appraisal Tool for Cross-Sectional Studies (AXIS) [33]. Publications were scored by both assessors independently by assigning a number from 1–100. The average of both scores were used to classify publications as high (80–100%), medium (60–79%), or low (< 60%).

### Data analysis

Data synthesis in this study involved narrative description and thematic presentation of studies in tables, charts and text [34]. This systematic review was designed to be exploratory thereby allowing for a review of the full scope of literature on this topic, irrespective of the type or quality of the study. This systematic review was not intended to compare efficacy across different models of care; the lack of uniform methodologies and standardization in service delivery make statistical meta-analysis and comparison between models of care not feasible.

## Results

### Characteristics of included literature

**Fig. 1:**
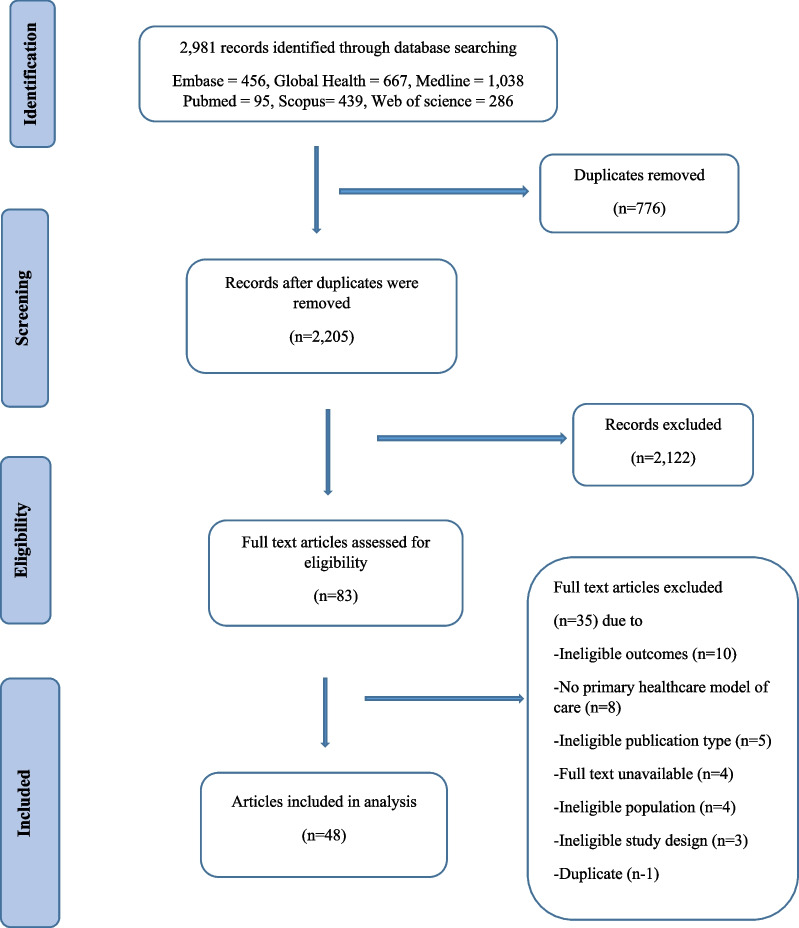
PRISMA chart showing study manuscript selection process

Initial database searches yielded 2,981 articles (Additional file 1: Appendix 1). After removing duplicates, the titles and abstracts of 2,205 articles were screened to ensure their content was related to the inclusion criteria for the study. The full text of eighty-three articles were reviewed and forty-eight articles were selected for inclusion in this review (Fig. 1).

Of the forty-eight included articles, thirty-three articles were rated as high quality, ten articles rated as medium, and five articles were rated as low quality. The majority of the publications included in this systematic review were from the latter half of the 1992–2020 date range; twenty-five (52%) were published after 2015, twenty-one (44%) were published between 2006–2015, and two (4%) were published between 1992–2005. Study designs included twenty-nine (60%) quantitative methods, thirteen (27%) qualitative methods, and six (12.5%) mixed qualitative and quantitative methods. Of the papers that were classified to have used quantitative and qualitative methods, sixteen were other peer-reviewed published studies describing programs led by humanitarian organizations. Additional file 2: Appendix 2 presents the characteristics of the studies included in this review.

**Fig. 2:**
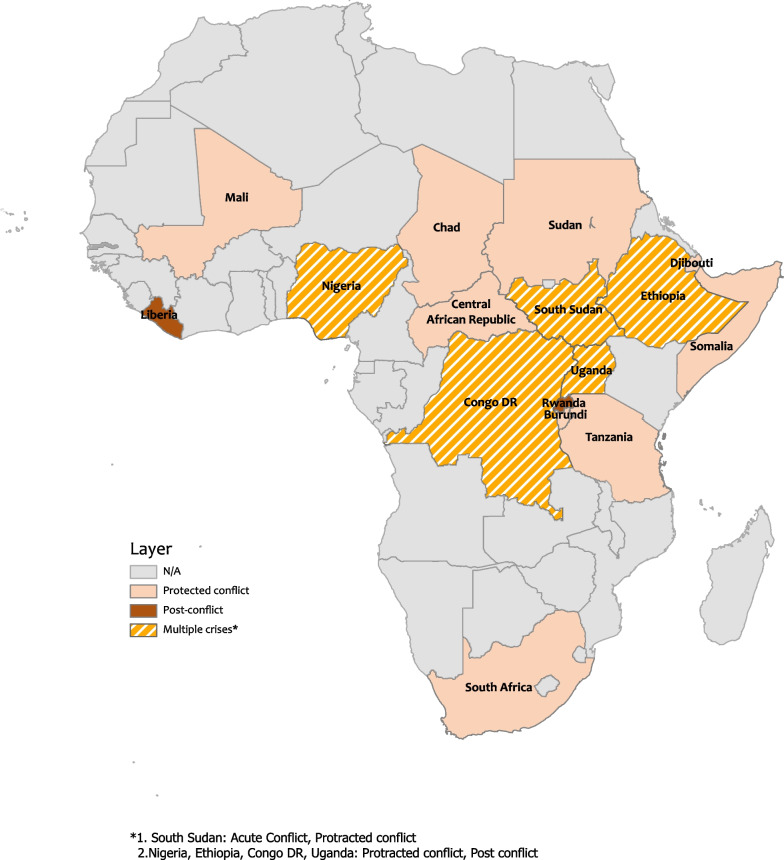
Geographical distribution of manuscripts included in the systematic review

Most of the studies were in countries experiencing protracted conflict (N = 36, 75%) (Fig. 2). Additional study settings included post-conflict countries (N = 10, 21%), and countries with acute conflict-affected settings (N = 2, 4%). Most studies included were from countries in Central and East Africa (Fig. 2). A range of institutions were reported to be implementing humanitarian health programs targeting conflict-affected communities; most papers noted institutions delivering primary healthcare services were international non-government organisations (INGOs) (N = 29) and governmental organizations (N = 28) (Additional file 2: Appendix 2). A full database of the forty-eight publications with data extracted is available in Additional file 3: Appendix 3.

### Models of care

A total of thirty-eight (79.2%) manuscripts reported the use of health centres/clinics as a model of healthcare delivery (Table 1). Twenty-one (44%) manuscripts reported the use of community-based (CB) models of care. Mobile clinics (N = 9, 19%), outreach (N = 9, 18%) and home visits (N = 3, 6%) were also reported in some manuscripts as seen in Table 2.

**Table 2:** Models of care and definitions

Model of care (publications)	Alternative names	Personnel used
Health facility based (N = 38) [36–42, 44–48, 50, 52, 57–64, 66, 67, 75–88]	Health centres, Clinics	Healthcare workers, Community health works (CHWs)
Community-based (N = 21) [37, 40, 41, 43–46, 49, 53–55, 58, 64, 66, 67, 69, 78, 81, 82, 85, 89]	Community health workers, village health teams, community health volunteers, lady health workers and community based distributors, Traditional birth attendants,	CHWs
Outreach (N = 9) [41, 45, 46, 51, 59, 63, 66, 83, 85]	Community outreach, fixed outreach site, aid post	Healthcare Workers, CHWs
Mobile clinic(N = 9) [36, 46, 51, 54, 63, 65, 78, 85, 90]	Mobile teams, mobile health centre, mobile health clinic, health camps,	Healthcare workers
Home-visit (N = 3) [58, 62, 88]	Household visits, house-to-house visits, tent-to-tent visits	Healthcare Workers, CHWs

### Services offered and human resources used

Most studies reported a range of services for communicable and non-communicable diseases offered by humanitarian organisations using one of the models of care presented in Table 2. It was observed that thirteen of forty-eight publications (Additional file 3: Appendix 3) reported the delivery of vertical interventions (single service) using their model of care as opposed to a comprehensive or integrated package of care involving more than two services as recommended by WHO [35]. Three papers reported the model of care used but did not specify the specific services offered [36–38]. Forty-four papers reported services delivered by government institutions and other organisations, while five papers reported services offered in government institutions only [39–43]. The services identified in these five papers reporting government institutions were services for: acute malnutrition, HIV, TB, immunisation, disease surveillance, malaria, acute respiratory infections, diarrhoea, SRH and health education.

While health facilities made use of skilled humanitarian personnel for health service delivery (e.g., doctors, nurses, midwives, clinical psychologist) (Table 3), community-based model of care tended to use those with less formal training (i.e., Community Health Workers [N = 17], traditional birth attendants [N = 2], lay persons [N = 4] and village health teams [N = 1]). Mobile clinics were reported to have doctors, nurses, midwives, and nursing assistants. Injury and trauma care, provided in health facilities and mobile clinics, was delivered by skilled personnel like doctors and nurses (Table 2). Services for non-communicable diseases were offered through health facilities and community-based models of care only, using nurses, doctors, medical assistants, nutritionist, and community health workers [44, 45]. The community health workers in the paper by Malembaka et al. [44], targeted and identified households with reported cases of diabetes, and hypertension.

**Table 3:**
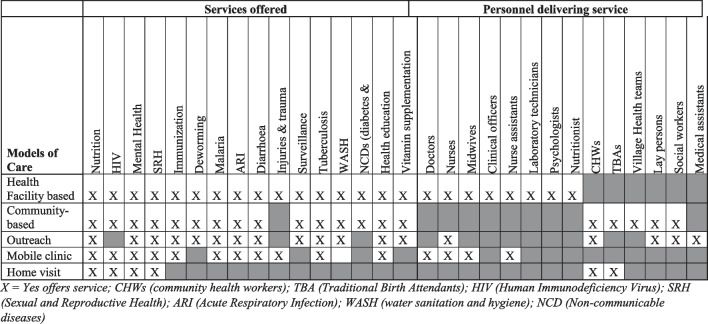
Services offered and personnel delivering service for each model of care

X = Yes offers service; CHWs (community health workers); TBA (Traditional Birth Attendants); HIV (Human Immunodeficiency Virus); SRH (Sexual and Reproductive Health); ARI (Acute Respiratory Infection); WASH (water sanitation and hygiene); NCD (Non-communicable diseases)

### Cost of services

In eighteen of forty-eight (38%) publications, it was reported whether the services offered by the institutions were free or provided for a cost. Of these publications, six reported some services being paid out-of-pocket at government and private owned facilities [37, 38, 44, 46–48]. Only three studies attempted to report the cost of services per beneficiaries of the interventions [42, 48, 49].

### Accessibility to services

In fourteen of the forty-eight (29%) manuscripts, aspects relating to accessibility of health services were addressed. Accessibility was either reported in walking distance between patient and health service [40, 47, 50, 51]; time required to reach service delivery point [36, 42, 43, 45, 52–56]; or transportation cost required to reach service providers [38, 45]. Walking distances to service delivery points reported ranged from 5 km and 7.2 km. One publication [46] mentioned distance as a major challenge to accessing reproductive, maternal, new born, child, adolescent health and nutrition services in Democratic Republic of Congo, but did not specify how many kilometres needed to be covered by individuals to access healthcare. In Uganda another group HIV voluntary testing and counselling, PMTCT, and ART services were located within accessible distances of IDP camps though specifics were not provided on how long IDPs were required to walk for to access the services [39]. Four manuscripts reported one hour walk to service delivery point [36, 52, 53], while three papers reported 1.5—2 h walking distance to service delivery point [43, 50, 54]. Murphy et al. reported many patients had to travel far distances for 4–5 h to access medications for diabetic care [57].

### Challenges to implementing models of care

Key challenges of providing primary healthcare to conflict-affected populations were mostly operational and logistical with the exception of work in Tanzania that reported challenges due to negative staff attitudes as evidenced by delays in registrations at health facilities and severe delays in issuing health cards to refugees [58]. Kidnappings [46, 59–61], gunshots and crossfire [39, 40, 46, 61–64], constant population displacement [39, 46, 62], poor roads [36, 50, 51, 53, 60, 65], lack of transportation [47, 50, 66], staff shortages [47, 64, 67] and shortages of medication and equipment [43, 63, 66, 67] were the main challenges reported. Robbery within refugee camps was also reported as a security issue faced in conflict-affected settings by one of the reviewed papers [58].

## Discussion

This systematic review examined the evidence describing primary healthcare delivery models used by humanitarian organisations and governments in conflict-affected settings in Africa. The provision of care in conflict-affected settings differ from routine service provision for multiple reasons. These include migration of the population into new areas with associated burden on existing health facilities, closure of some health facilities, additional burden of disease/ injuries due to conflict and limitations of population/ health worker movement due to lockdowns/curfews. In these settings, often provision of services is by humanitarian organisations rather than government due to their reduced capacity for health service delivery during conflicts [5, 68]. This review focuses on the existing evidence about how healthcare is delivered in conflict-affected settings. Of the forty-eight manuscripts included in the review, sixteen were other peer-reviewed published studies describing interventions led by humanitarian organisations as program response rather than findings from primary research studies. This reflects the gap in evidence-based research in conflict-affected settings. There is a recent increase in the number of publications in conflict-affected settings, with most being published in the last five years. This may be due to the global call for more research addressing the evidence gap in humanitarian health as highlighted by Kohrt et al. [68].

Many of the studies included in this review were cross sectional studies (n = 15) with only one randomised controlled trial [69] and one economic evaluation study [48]. It is critical that studies make use of robust methods in research on healthcare delivery models in conflict-affected settings to provide information on how to best ensure integration of services, and delivery of high quality and cost-effective care.

### Choice of models of care

Understanding how organisations chose models of care in conflict-affected settings is of critical importance to ensure quality and value for money. However, we were unable to find any papers in this review which explored the choice of care model. Several considerations should guide the use of any model of care in conflict-affected settings. McGowan et al. 2020 [17] suggests that analysis around frequency of visits to communities and the package of services offered should underpin the choice of a model of care in such settings. Other factors like donor and governmental priorities, contextual evidence, need, and insecurity may significantly influence the choice of each model of care. Health facility-based, and community-based models were the leading models of primary healthcare delivery reported; this may be because they may be cheaper and more sustainable in these difficult settings.

Although the evidence on the use of community-based model of care such as community health workers in these settings is limited, strategies to improve service delivery and access to services is needed. In conflict-affected settings, there may be opportunities to coordinate care/service delivery to meet the minimum standards for quality as outlined by Sphere guidelines [70]. Whilst access to quality healthcare is a human right and should be a standard for all health services, populations living in conflict-affected settings experience particular health stressors (disrupted healthcare, poor living conditions, mental health problems pre/post displacements) and may need models of care that can support the additional needs of this population.

### The relevance of understanding the landscape of implementing institutions

Twelve publications reported health care provided by national non-governmental organizations and twenty-nine by international non-governmental organizations (Additional file 2: Appendix 2). Townes et al. [5] describes the humanitarian ecosystem as one marked by the usual influx of many humanitarian organizations who most often fight for limited resources and relevance in conflict-affected settings at the expense of quality healthcare [5]. Faced with such, it is important to reinforce coordination between these implementing institutions to avoid duplication of efforts and maximized the use of limited resources for the benefit of the affected populations [71]. Coordination could be improved when government and humanitarian organizations have complete information of each institution’s interventions. Publications included in this systematic review did not provide enough data/information to the specific context of the conflict-affected setting and the roles of NGOs as compared to government agencies in each case (Additional file 2: Appendix 2). Whilst the humanitarian health cluster system frequently use the 5Ws (Who does, What, Where, When, and to Whom) mapping matrix to aid in coordination, it does not include information on the modalities (by Which means?) organizations use to reach the affected populations. Information on which models of care are used in what localities to deliver primary healthcare in conflict-affected settings might reinforce steps needed to ensure better coordination, predictability, and sustainability.

### Moving towards integrated services in health responses within conflict-affected settings

The integration of health services (delivery and management of health services which include health promotion, disease prevention, diagnosis, treatment, disease management, rehabilitation and palliative care services) [35], yields reduced costs of service delivery, avoids duplication of services, and maximizes opportunities to build on collaborations in health systems especially in resource-constrained settings like conflict-affected settings of Africa. Also, integrated care better addresses preventive medicine and continuity of care after the acute phase of a crisis. Disease specific programs and responses offer limited opportunities to prevent and treat other existing health conditions in difficult to reach populations in conflict-affected settings [72]. The aforementioned disadvantages of stand-alone interventions could be averted through integration of health services.

Provision of an integrated range of services was observed in this review, though there were few examples, and most were health facility-based. The provision of stand-alone services using mobile clinics, community-based or outreach models of care was noted in thirteen publications. In such difficult settings, it might be more efficient to provide an integrated package of services. Druetz [73] reported the lack of primary healthcare integration as being one of the main limits to programs’ efficacy in low- and middle-income countries. As most conflict-affected settings are increasingly becoming protracted and faced with an increasing double burden of disease from non-communicable diseases, communicable diseases, and pandemics like COVID-19, it might be rational, cost effective, less time consuming and less risky, for services to be delivered using an integrated approach. The vertical approach (stand-alone) to service delivery should be of concern to the international humanitarian community and donors. It is necessary for donors to ensure models of care that are being funded are less fragmented while being timely, safe and of good quality to communities. WHO highlights the importance of integrated health services in meeting the needs of communities throughout their life course [35].

The papers included in this study reported communicable diseases being offered by government institutions while services reported for humanitarian organisations included both communicable and non-communicable diseases. This could be because only four papers from the forty-eight publications reported services of interventions carried out by government institutions. This highlights an important gap in literature and the need for publication of evaluations of governmental interventions in conflict-affected settings.

### Rethinking human resources used in conflict-affected settings

The availability and accessibility of skilled human resources to provide primary healthcare in conflict-affected settings is often limited for various reasons. These include but are not limited to insufficient pre-conflict health care worker training and compounded by increased need for healthcare due to conflict related injury and illness, difficulty in health care worker mobility due to insecurity and poor transport infrastructure in conflict-settings. Therefore, delivering healthcare differently using non-specialist human resources such as lay community health workers may be an option to help enhance the availability of and access to primary healthcare in conflict-affected contexts and may offer long term solutions to staff shortages/unavailability. The studies in this review reported the use of both medical and non-medical human resources for health in conflict-affected settings. With the exception of injuries and trauma care, all other fifteen services reported in this review were provided at the primary care level and included community health workers. Our findings corroborate existing literature which found that community health workers were reported to provide critical emergency care and a range of health services in acute and protracted conflict-affected settings [18]. Rigorous studies on service delivery using community based non-medical staff such as community health workers in conflict-affected settings needs to be conducted. Keeping in mind the challenges of ensuring rigour in conflict-affected settings owing to their complexities, research which uses implementation approaches should be strongly considered by researchers.

### Challenges of provision of care

The model of care chosen is likely to be influenced by multiple factors as highlighted above, but the choice of model may have an impact on the level of access, range of services, health worker experience, training and seniority, time resources available for each consultation, referral mechanisms to specialist care, all of which contribute to overall quality of care. Additionally humanitarian health responses in conflict-affected settings are frequently challenged by life threatening security incidents or operational barriers as reported by an earlier review of challenges in humanitarian care [74]. Organisations have an ethical obligation to optimize safety for employees working in conflict-affected settings and affected populations and quality of care provided is likely to be higher when healthcare worker comfort and security are optimized. Therefore, safety considerations will influence the choice of delivery models and choice of model may involve a balance between safety of staff and population, maximizing access, ensuring a range of services delivered, and overall quality of care. Overall, we feel this is an understudied area and there is a need for more research to be conducted on measuring quality of care and optimizing health-related outcomes for different models of care in conflict-affected settings.

## Limitations

This study has a number of limitations that should be considered. We only found thirty-three high quality papers. Therefore, we also included five low quality studies and other peer-reviewed published studies describing programs led by humanitarian organisations (Additional file 3: Appendix 3). Further, it was not possible to do a meta-analysis to determine the effect of each model on health outcomes due to the heterogeneity in methodologies and lack of quantitative data on outcomes. Although we did not evaluate the effect each model of care had on health outcomes in conflict-affected settings, this research provides the foundation for future research in primary healthcare delivery in conflict-affected settings. Also, limiting our search criteria to conflict-affected settings in Africa will have impacted the number of included publications in the study, and may have also reduced the generalisability of the findings. However, the pre-conflict existing healthcare challenges due to high burden of disease and low resources that compound health systems in Africa necessitates that context-specific evidence drives reforms and programming that are practical to the African setting. Further, due to language limitations, we only reviewed papers published in English which may have reduced the number of publications included in this review.

### Gaps in literature/ challenge of research in conflict-affected settings

Evidence on the cost and cost effectiveness of different delivery models is paramount to influencing funding decisions in conflict-affected settings, yet only three publications included in this review reported on cost. Given that cost is a justification for model selection by some organizations, further information around the cost per beneficiary is needed. Economic evaluation studies are needed to guide decisions around the most effective use of resources to optimize quality primary healthcare delivery in this unique context. The cost to organisations providing care is part of the consideration for choice of model; however, any cost analysis should consider the cost to the beneficiary. This would include out-of-pocket expenses for care and for medications, but also for transportation.

Also, it is important to document the coverage of services by model of care to identify gaps in populations reached and/or services delivered. Lastly, future research being published in conflict-affected settings should report on health outcomes resulting from the use of primary healthcare models. Longitudinal and randomized controlled studies aimed at improving quality and access to healthcare in conflict settings would provide higher quality evidence than observational studies, albeit expensive to conduct. However, this may be difficult, as they need to be carefully designed to ensure ethical conduct in challenging circumstances with vulnerable populations.

## Conclusion

There is a very limited published evidence base on which models of care are used to deliver primary healthcare in conflict-affected settings in Africa. Further, there were a few publications which outlined a rational/ process for choice of model of care by organisations working in conflict-affected settings. While evidence should guide the selection of which model of care to use at any time in conflict-affected settings, staff/population safety and accessibility of services to communities are currently and will continue to be key factors in the decision-making process.

## Supplementary Information

**Additional file 1.** Systematic review search strategy.

**Additional file 2.** Characteristics of included literature.

**Additional file 3.** Systematic review results with man characteristics.

## Data Availability

The data generated from this review is provided in the supplementary information files.

## Abbreviations

PHC: Primary healthcare
CB: Community based
CHW: Community health workers
IDPs: Internally displaced persons
INGOs: International non-governmental organisation
NNGO: National non-governmental organisation
FBO: Faith-based organisation
CBO: Community-based organisation
UN: United Nation
