# Supplementary material for: Primary healthcare delivery models in African conflict-affected settings: a systematic review
Source: Confl Health. 2023 Jul 15;17:34. doi: 10.1186/s13031-023-00533-w (PMC10349495; doi:10.1186/s13031-023-00533-w)
Supplement: Supplementary file 1 — Additional file 1. Systematic review search strategy. [file 13031_2023_533_MOESM1_ESM.docx]

## **APPENDIX 1**

## **Search Strategy and results obtained from data bases search for Systematic Review**

## **MEDLINE (n= 1038)** **from 1988 to 2020 week 50 searched and exported on the 16/12/2020**

| 1 | (primary health care or primary prevention or comprehensive health care Primary care Service delivery or Mobile clinic* or mobile service or ambulatory care facilities or mobile health unit or outpatient clinic* or Community health worker* or village health teams or Health centre* or community clinic* or maternal health center* or Health facilit* or Hospital*).mp. [mp=title, abstract, heading word, drug trade name, original title, device manufacturer, drug manufacturer, device trade name, keyword, floating subheading word, candidate term word] | 2629190 |
| --- | --- | --- |
| 2 | (Conflict affected* or Humanitarian*or Civil war or Relief work or internally Displaced person* or refugee*).mp. [mp=title, abstract, heading word, drug trade name, original title, device manufacturer, drug manufacturer, device trade name, keyword, floating subheading word, candidate term word] | 17040 |
| 3 | (((Burundi or Rwanda or Kenya or South Sudan or Tanzania or Uganda or Cameroon or central Africa republic or Sao Tome) and Principe) or Djibouti or Eritrea or Ethiopia or Somalia or Comoros or Madagascar or Mauritius or Algeria or Egypt or Libya or Morocco or Sudan or Tunisia or Angola or Lesotho or Malawi or Mozambique or Namibia or south Africa or Swaziland or Zambia or Zimbabwe or Benin or Burkina faso or Ivory Cost or Gambia or Ghana or guinea or guinea Bissau or Liberia or Mali or Mauritania or Niger or Nigeria or sierra leone or western Sahara).mp. [mp=title, abstract, heading word, drug trade name, original title, device manufacturer, drug manufacturer, device trade name, keyword, floating subheading word, candidate term word] | 337172 |
| 4 | exp primary health care/ | 167263 |
| 5 | exp primary medical care/ | 107677 |
| 6 | exp health care delivery/ | 3265048 |
| 7 | 4 or 5 or 6 | 3265048 |
| 8 | 1 or 7 | 5007863 |
| 9 | (Africa* or Sub-Saharan Africa or West Africa or East Africa).mp. [mp=title, abstract, heading word, drug trade name, original title, device manufacturer, drug manufacturer, device trade name, keyword, floating subheading word, candidate term word] | 343298 |
| 10 | 3 or 9 | 583239 |
| 11 | 2 and 8 and 10 | 1038 |

1. **Embase (n= 456) from 1988 to 2020 week 50**

| **#** | **Query** | **Results from 16 Dec 2020** |
| --- | --- | --- |
| 1 | exp primary health care/ | 167,263 |
| 2 | exp family medicine/ | 10,983 |
| 3 | exp primary medical care/ | 107,677 |
| 4 | (Primary health care or primary prevention or comprehensive health care Primary care Service delivery or Mobile clinic* or mobile service or ambulatory care facilities or mobile health unit or outpatient clinic* or Community health worker* or village health teams or Health centre* or community clinic* or maternal health center* or Health facility* or Hospital*).mp. [mp=title, abstract, heading word, drug trade name, original title, device manufacturer, drug manufacturer, device trade name, keyword, floating subheading word, candidate term word] | 2,622,545 |
| 5 | (Conflict affected* or Humanitarian*or Civil war or Relief work or internally Displaced person* or refugee*).mp. [mp=title, abstract, heading word, drug trade name, original title, device manufacturer, drug manufacturer, device trade name, keyword, floating subheading word, candidate term word] | 17,040 |
| 6 | (Africa* or Sub-Saharan Africa or West Africa or East Africa or Central Africa).mp. [mp=title, abstract, heading word, drug trade name, original title, device manufacturer, drug manufacturer, device trade name, keyword, floating subheading word, candidate term word] | 343,298 |
| 7 | (((Burundi or Rwanda or Kenya or South Sudan or Tanzania or Uganda or Cameroon or central Africa republic or Sao Tome) and Principe) or Djibouti or Eritrea or Ethiopia or Somalia or Comoros or Madagascar or Mauritius or Algeria or Egypt or Libya or Morocco or Sudan or Tunisia or Angola or Lesotho or Malawi or Mozambique or Namibia or south Africa or Swaziland or Zambia or Zimbabwe or Benin or Burkina faso or Ivory Cost or Gambia or Ghana or guinea or guinea Bissau or Liberia or Mali or Mauritania or Niger or Nigeria or sierra leone or western Sahara or Democratic Republic of Congo).mp. [mp=title, abstract, heading word, drug trade name, original title, device manufacturer, drug manufacturer, device trade name, keyword, floating subheading word, candidate term word] | 340,950 |
| 8 | 6 or 7 | 585,804 |
| 9 | 1 or 2 or 3 or 4 | 2,704,271 |
| 10 | 8 and 9 and 5 | 456 |

1. **Global Health/ebscohost (n=667) from 1988 to 2020 downloaded the 16/12/2020**

| S1 | Primary health care or primary prevention or comprehensive health care or Primary care Service delivery or Mobile clinic* or mobile service or ambulatory care facilities or mobile health unit or outpatient clinic* or Community health worker* or village health teams or Health centre* or community clinic* or maternal health center* or Health facility* or Hospital*or family medicine or primary care | 112,510 |
| --- | --- | --- |
| S2 | Conflict affected* or conflict or Humanitarian*or Civil war or Relief work or internally Displaced person* or refugee* | 30,659 |
| S3 | Africa* or Sub-Saharan Africa or West Africa or East Africa or Central Africa | 278,615 |
| S4 | (Burundi or Rwanda or Kenya or South Sudan or Tanzania or Uganda or Cameroon or central Africa republic or Sao Tome and Principe or Djibouti or Eritrea or Ethiopia or Somalia or Comoros or Madagascar or Mauritius or Algeria or Egypt or Libya or Morocco or Sudan or Tunisia or Angola or Lesotho or Malawi or Mozambique or Namibia or south Africa or Swaziland or Zambia or Zimbabwe or Benin or Burkina faso or Ivory Cost or Gambia or Ghana or guinea or guinea Bissau or Liberia or Mali or Mauritania or Niger or Nigeria or sierra leone or western Sahara or Democratic Republic of Congo) | 303,823 |
| S5 | S1 AND S2 AND S3 AND S4 | 667 |

1. **Scopus ( search done on the 16/12/2020 and exported to endnote same day**

| #1, un-filtered search | ( ALL ( "Primary health care" ) OR "family medicine" OR "primary prevention" OR "comprehensive health care" OR "Primary care Service delivery" OR "Mobile clinic*" OR "mobile service" OR "ambulatory care facilities" OR "mobile health unit" OR "outpatient clinic*" OR "Community health worker*" OR "village health teams" OR "Health centre*" OR "community clinic*" OR "maternal health center*" OR "Health facility*" OR "Hospital*or family medicine" OR "primary care" ) AND ( ALL ( "Conflict affected*" ) OR "conflict" OR "Humanitarian*" OR "Civil war" OR "Relief work" OR "internally Displaced person*" OR "refugee*" ) AND ( ( ALL ( africa* ) OR "Sub-Saharan Africa" OR "West Africa" OR "East Africa" OR "Central Africa" ) OR ( ALL ( burundi ) OR rwanda OR kenya OR "South Sudan" OR tanzania OR uganda OR cameroon OR "central Africa republic" OR "Sao Tome" AND principe OR djibouti OR eritrea OR ethiopia OR somalia OR comoros OR madagascar OR mauritius OR algeria OR egypt OR libya OR morocco OR sudan OR tunisia OR angola OR lesotho OR malawi OR mozambique OR namibia OR "south Africa" OR swaziland OR zambia OR zimbabwe OR benin OR "Burkina faso" OR "Ivory Cost" OR gambia OR ghana OR guinea OR "guinea Bissau" OR liberia OR mali OR mauritania OR niger OR nigeria OR "sierra leone" OR "western Sahara" OR "Democratic Republic of Congo" ) ) | 19,506 |
| --- | --- | --- |
| #2, filtered search | ( ALL ( "Primary health care" ) OR "family medicine" OR "primary prevention" OR "comprehensive health care" OR "Primary care Service delivery" OR "Mobile clinic*" OR "mobile service" OR "ambulatory care facilities" OR "mobile health unit" OR "outpatient clinic*" OR "Community health worker*" OR "village health teams" OR "Health centre*" OR "community clinic*" OR "maternal health center*" OR "Health facility*" OR "Hospital*or family medicine" OR "primary care" ) AND ( ALL ( "Conflict affected*" ) OR "conflict" OR "Humanitarian*" OR "Civil war" OR "Relief work" OR "internally Displaced person*" OR "refugee*" ) AND ( ( ALL ( africa* ) OR "Sub-Saharan Africa" OR "West Africa" OR "East Africa" OR "Central Africa" ) OR ( ALL ( burundi ) OR rwanda OR kenya OR "South Sudan" OR tanzania OR uganda OR cameroon OR "central Africa republic" OR "Sao Tome" AND principe OR djibouti OR eritrea OR ethiopia OR somalia OR comoros OR madagascar OR mauritius OR algeria OR egypt OR libya OR morocco OR sudan OR tunisia OR angola OR lesotho OR malawi OR mozambique OR namibia OR "south Africa" OR swaziland OR zambia OR zimbabwe OR benin OR "Burkina faso" OR "Ivory Cost" OR gambia OR ghana OR guinea OR "guinea Bissau" OR liberia OR mali OR mauritania OR niger OR nigeria OR "sierra leone" OR "western Sahara" OR "Democratic Republic of Congo" ) ) AND ( LIMIT-TO ( PUBYEAR , 2021 ) OR LIMIT-TO ( PUBYEAR , 2020 ) OR LIMIT-TO ( PUBYEAR , 2019 ) OR LIMIT-TO ( PUBYEAR , 2018 ) OR LIMIT-TO ( PUBYEAR , 2017 ) OR LIMIT-TO ( PUBYEAR , 2016 ) OR LIMIT-TO ( PUBYEAR , 2015 ) OR LIMIT-TO ( PUBYEAR , 2014 ) OR LIMIT-TO ( PUBYEAR , 2013 ) OR LIMIT-TO ( PUBYEAR , 2012 ) OR LIMIT-TO ( PUBYEAR , 2011 ) OR LIMIT-TO ( PUBYEAR , 2010 ) OR LIMIT-TO ( PUBYEAR , 2009 ) OR LIMIT-TO ( PUBYEAR , 2008 ) OR LIMIT-TO ( PUBYEAR , 2007 ) OR LIMIT-TO ( PUBYEAR , 2006 ) OR LIMIT-TO ( PUBYEAR , 2005 ) OR LIMIT-TO ( PUBYEAR , 2004 ) OR LIMIT-TO ( PUBYEAR , 2003 ) OR LIMIT-TO ( PUBYEAR , 2002 ) OR LIMIT-TO ( PUBYEAR , 2001 ) OR LIMIT-TO ( PUBYEAR , 2000 ) OR LIMIT-TO ( PUBYEAR , 1999 ) OR LIMIT-TO ( PUBYEAR , 1998 ) OR LIMIT-TO ( PUBYEAR , 1997 ) OR LIMIT-TO ( PUBYEAR , 1996 ) OR LIMIT-TO ( PUBYEAR , 1995 ) OR LIMIT-TO ( PUBYEAR , 1994 ) OR LIMIT-TO ( PUBYEAR , 1993 ) OR LIMIT-TO ( PUBYEAR , 1992 ) OR LIMIT-TO ( PUBYEAR , 1991 ) OR LIMIT-TO ( PUBYEAR , 1990 ) OR LIMIT-TO ( PUBYEAR , 1989 ) OR LIMIT-TO ( PUBYEAR , 1988 ) ) AND ( LIMIT-TO ( PUBSTAGE , "final" ) ) AND ( LIMIT-TO ( DOCTYPE , "ar" ) ) AND ( LIMIT-TO ( SUBJAREA , "MEDI" ) OR LIMIT-TO ( SUBJAREA , "SOCI" ) OR LIMIT-TO ( SUBJAREA , "PSYC" ) OR LIMIT-TO ( SUBJAREA , "NURS" ) OR LIMIT-TO ( SUBJAREA , "ARTS" ) ) AND ( LIMIT-TO ( LANGUAGE , "English" ) ) AND ( EXCLUDE ( EXACTKEYWORD , "United States" ) OR EXCLUDE ( EXACTKEYWORD , "Intimate Partner Violence" ) OR EXCLUDE ( EXACTKEYWORD , "Sex Difference" ) OR EXCLUDE ( EXACTKEYWORD , "Perception" ) OR EXCLUDE ( EXACTKEYWORD , "African Americans" ) OR EXCLUDE ( EXACTKEYWORD , "Hispanic" ) ) AND ( EXCLUDE ( SUBJAREA , "BIOC" ) OR EXCLUDE ( SUBJAREA , "BUSI" ) OR EXCLUDE ( SUBJAREA , "CENG" ) OR EXCLUDE ( SUBJAREA , "COMP" ) OR EXCLUDE ( SUBJAREA , "EART" ) ) AND ( EXCLUDE ( SUBJAREA , "IMMU" ) OR EXCLUDE ( SUBJAREA , "SOCI" ) OR EXCLUDE ( SUBJAREA , "ENVI" ) OR EXCLUDE ( SUBJAREA , "NEUR" ) OR EXCLUDE ( SUBJAREA , "PHAR" ) OR EXCLUDE ( SUBJAREA , "ECON" ) OR EXCLUDE ( SUBJAREA , "AGRI" ) OR EXCLUDE ( SUBJAREA , "ENGI" ) OR EXCLUDE ( SUBJAREA , "ENER" ) OR EXCLUDE ( SUBJAREA , "MATH" ) OR EXCLUDE ( SUBJAREA , "VETE" ) OR EXCLUDE ( SUBJAREA , "DENT" ) OR EXCLUDE ( SUBJAREA , "DECI" ) ) AND ( EXCLUDE ( AFFILCOUNTRY , "United States" ) OR EXCLUDE ( AFFILCOUNTRY , "United Kingdom" ) OR EXCLUDE ( AFFILCOUNTRY , "Canada" ) OR EXCLUDE ( AFFILCOUNTRY , "Australia" ) OR EXCLUDE ( AFFILCOUNTRY , "Switzerland" ) OR EXCLUDE ( AFFILCOUNTRY , "Netherlands" ) OR EXCLUDE ( AFFILCOUNTRY , "Germany" ) OR EXCLUDE ( AFFILCOUNTRY , "Sweden" ) OR EXCLUDE ( AFFILCOUNTRY , "Italy" ) OR EXCLUDE ( AFFILCOUNTRY , "India" ) OR EXCLUDE ( AFFILCOUNTRY , "Norway" ) OR EXCLUDE ( AFFILCOUNTRY , "France" ) OR EXCLUDE ( AFFILCOUNTRY , "Spain" ) OR EXCLUDE ( AFFILCOUNTRY , "Belgium" ) OR EXCLUDE ( AFFILCOUNTRY , "China" ) OR EXCLUDE ( AFFILCOUNTRY , "Pakistan" ) OR EXCLUDE ( AFFILCOUNTRY , "Iran" ) OR EXCLUDE ( AFFILCOUNTRY , "Israel" ) OR EXCLUDE ( AFFILCOUNTRY , "Brazil" ) OR EXCLUDE ( AFFILCOUNTRY , "Denmark" ) OR EXCLUDE ( AFFILCOUNTRY , "Lebanon" ) OR EXCLUDE ( AFFILCOUNTRY , "Nepal" ) OR EXCLUDE ( AFFILCOUNTRY , "New Zealand" ) OR EXCLUDE ( AFFILCOUNTRY , "Finland" ) OR EXCLUDE ( AFFILCOUNTRY , "Jordan" ) OR EXCLUDE ( AFFILCOUNTRY , "Japan" ) OR EXCLUDE ( AFFILCOUNTRY , "Thailand" ) OR EXCLUDE ( AFFILCOUNTRY , "Bangladesh" ) OR EXCLUDE ( AFFILCOUNTRY , "South Korea" ) OR EXCLUDE ( AFFILCOUNTRY , "Ireland" ) OR EXCLUDE ( AFFILCOUNTRY , "Saudi Arabia" ) OR EXCLUDE ( AFFILCOUNTRY , "Afghanistan" ) OR EXCLUDE ( AFFILCOUNTRY , "Turkey" ) OR EXCLUDE ( AFFILCOUNTRY , "Malaysia" ) OR EXCLUDE ( AFFILCOUNTRY , "Hong Kong" ) OR EXCLUDE ( AFFILCOUNTRY , "Iraq" ) OR EXCLUDE ( AFFILCOUNTRY , "Portugal" ) OR EXCLUDE ( AFFILCOUNTRY , "Colombia" ) OR EXCLUDE ( AFFILCOUNTRY , "Palestine" ) OR EXCLUDE ( AFFILCOUNTRY , "Indonesia" ) OR EXCLUDE ( AFFILCOUNTRY , "Mexico" ) OR EXCLUDE ( AFFILCOUNTRY , "Taiwan" ) OR EXCLUDE ( AFFILCOUNTRY , "Sri Lanka" ) OR EXCLUDE ( AFFILCOUNTRY , "Greece" ) OR EXCLUDE ( AFFILCOUNTRY , "Austria" ) OR EXCLUDE ( AFFILCOUNTRY , "Peru" ) OR EXCLUDE ( AFFILCOUNTRY , "Russian Federation" ) OR EXCLUDE ( AFFILCOUNTRY , "Cambodia" ) OR EXCLUDE ( AFFILCOUNTRY , "Singapore" ) OR EXCLUDE ( AFFILCOUNTRY , "Chile" ) OR EXCLUDE ( AFFILCOUNTRY , "Philippines" ) OR EXCLUDE ( AFFILCOUNTRY , "Qatar" ) OR EXCLUDE ( AFFILCOUNTRY , "Viet Nam" ) OR EXCLUDE ( AFFILCOUNTRY , "Poland" ) OR EXCLUDE ( AFFILCOUNTRY , "United Arab Emirates" ) OR EXCLUDE ( AFFILCOUNTRY , "Serbia" ) OR EXCLUDE ( AFFILCOUNTRY , "Yemen" ) OR EXCLUDE ( AFFILCOUNTRY , "Myanmar" ) OR EXCLUDE ( AFFILCOUNTRY , "Oman" ) OR EXCLUDE ( AFFILCOUNTRY , "Ukraine" ) OR EXCLUDE ( AFFILCOUNTRY , "Romania" ) OR EXCLUDE ( AFFILCOUNTRY , "Bahrain" ) OR EXCLUDE ( AFFILCOUNTRY , "Croatia" ) OR EXCLUDE ( AFFILCOUNTRY , "Luxembourg" ) OR EXCLUDE ( AFFILCOUNTRY , "Slovakia" ) OR EXCLUDE ( AFFILCOUNTRY , "Argentina" ) OR EXCLUDE ( AFFILCOUNTRY , "Estonia" ) OR EXCLUDE ( AFFILCOUNTRY , "Namibia" ) OR EXCLUDE ( AFFILCOUNTRY , "Georgia" ) OR EXCLUDE ( AFFILCOUNTRY , "Iceland" ) OR EXCLUDE ( AFFILCOUNTRY , "Kuwait" ) OR EXCLUDE ( AFFILCOUNTRY , "Kazakhstan" ) OR EXCLUDE ( AFFILCOUNTRY , "Federated States of Micronesia" ) OR EXCLUDE ( AFFILCOUNTRY , "Kyrgyzstan" ) OR EXCLUDE ( AFFILCOUNTRY , "Macao" ) OR EXCLUDE ( AFFILCOUNTRY , "Panama" ) OR EXCLUDE ( AFFILCOUNTRY , "Tunisia" ) OR EXCLUDE ( AFFILCOUNTRY , "Anguilla" ) OR EXCLUDE ( AFFILCOUNTRY , "Costa Rica" ) OR EXCLUDE ( AFFILCOUNTRY , "Czech Republic" ) OR EXCLUDE ( AFFILCOUNTRY , "Guatemala" ) OR EXCLUDE ( AFFILCOUNTRY , "Hungary" ) OR EXCLUDE ( AFFILCOUNTRY , "Syrian Arab Republic" ) OR EXCLUDE ( AFFILCOUNTRY , "Bolivia" ) OR EXCLUDE ( AFFILCOUNTRY , "Ecuador" ) OR EXCLUDE ( AFFILCOUNTRY , "Laos" ) OR EXCLUDE ( AFFILCOUNTRY , "Uruguay" ) OR EXCLUDE ( AFFILCOUNTRY , "Bulgaria" ) OR EXCLUDE ( AFFILCOUNTRY , "Libyan Arab Jamahiriya" ) OR EXCLUDE ( AFFILCOUNTRY , "Slovenia" ) OR EXCLUDE ( AFFILCOUNTRY , "Fiji" ) OR EXCLUDE ( AFFILCOUNTRY , "Albania" ) OR EXCLUDE ( AFFILCOUNTRY , "Barbados" ) OR EXCLUDE ( AFFILCOUNTRY , "Bhutan" ) OR EXCLUDE ( AFFILCOUNTRY , "Bosnia and Herzegovina" ) OR EXCLUDE ( AFFILCOUNTRY , "Brunei Darussalam" ) OR EXCLUDE ( AFFILCOUNTRY , "Cyprus" ) OR EXCLUDE ( AFFILCOUNTRY , "Moldova" ) OR EXCLUDE ( AFFILCOUNTRY , "New Caledonia" ) OR EXCLUDE ( AFFILCOUNTRY , "Timor-Leste" ) OR EXCLUDE ( AFFILCOUNTRY , "Armenia" ) OR EXCLUDE ( AFFILCOUNTRY , "Jamaica" ) OR EXCLUDE ( AFFILCOUNTRY , "Lithuania" ) OR EXCLUDE ( AFFILCOUNTRY , "Malta" ) OR EXCLUDE ( AFFILCOUNTRY , "Nicaragua" ) OR EXCLUDE ( AFFILCOUNTRY , "Solomon Islands" ) OR EXCLUDE ( AFFILCOUNTRY , "Swaziland" ) OR EXCLUDE ( AFFILCOUNTRY , "Trinidad and Tobago" ) OR EXCLUDE ( AFFILCOUNTRY , "Belarus" ) OR EXCLUDE ( AFFILCOUNTRY , "El Salvador" ) OR EXCLUDE ( AFFILCOUNTRY , "Puerto Rico" ) OR EXCLUDE ( AFFILCOUNTRY , "Samoa" ) OR EXCLUDE ( AFFILCOUNTRY , "Azerbaijan" ) OR EXCLUDE ( AFFILCOUNTRY , "Belize" ) OR EXCLUDE ( AFFILCOUNTRY , "Cuba" ) OR EXCLUDE ( AFFILCOUNTRY , "Undefined" ) ) | 439 |
|  |  |  |

1. **Web of science (n-286) search done on the 16/12/2020**

| **1** | TS=(("Primary health care" ) OR "family medicine" OR "primary prevention" OR "comprehensive health care" OR "Primary care Service delivery" OR "Mobile clinic*" OR "mobile service" OR "ambulatory care facilities" OR "mobile health unit" OR "outpatient clinic*" OR "Community health worker*" OR "village health teams" OR "Health centre*" OR "community clinic*" OR "maternal health center*" OR "Health facility*" OR "Hospital*or family medicine" OR "primary care" )  Indexes=SCI-EXPANDED, SSCI, A&HCI, CPCI-S, CPCI-SSH, BKCI-S, BKCI-SSH, ESCI, CCR-EXPANDED, IC Timespan=1988-2020 | [226,015](https://apps.webofknowledge.com/summary.do?product=WOS&doc=1&qid=17&SID=F2oLAMrfER9AbpfnOh2&search_mode=AdvancedSearch&update_back2search_link_param=yes) |
| --- | --- | --- |
| **2** | TS=( ALL ( "Conflict affected*" ) OR "conflict" OR "Humanitarian*" OR "Civil war" OR "Relief work" OR "internally Displaced person*" OR "refugee*" )  Indexes=SCI-EXPANDED, SSCI, A&HCI, CPCI-S, CPCI-SSH, BKCI-S, BKCI-SSH, ESCI, CCR-EXPANDED, IC Timespan=1988-2020 | [269,906](https://apps.webofknowledge.com/summary.do?product=WOS&doc=1&qid=19&SID=F2oLAMrfER9AbpfnOh2&search_mode=AdvancedSearch&update_back2search_link_param=yes) |
| **3** | TS= ( ( ALL ( africa* ) OR "Sub-Saharan Africa" OR "West Africa" OR "East Africa" OR "Central Africa" ) OR ( ALL ( burundi ) OR rwanda OR kenya OR "South Sudan" OR tanzania OR uganda OR cameroon OR "central Africa republic" OR "Sao Tome" AND principe OR djibouti OR eritrea OR ethiopia OR somalia OR comoros OR madagascar OR mauritius OR algeria OR egypt OR libya OR morocco OR sudan OR tunisia OR angola OR lesotho OR malawi OR mozambique OR namibia OR "south Africa" OR swaziland OR zambia OR zimbabwe OR benin OR "Burkina faso" OR "Ivory Cost" OR gambia OR ghana OR guinea OR "guinea Bissau" OR liberia OR mali OR mauritania OR niger OR nigeria OR "sierra leone" OR "western Sahara" OR "Democratic Republic of Congo" ) ) Indexes=SCI-EXPANDED, SSCI, A&HCI, CPCI-S, CPCI-SSH, BKCI-S, BKCI-SSH, ESCI, CCR-EXPANDED, IC Timespan=1988-2020 | [725,059](https://apps.webofknowledge.com/summary.do?product=WOS&doc=1&qid=20&SID=F2oLAMrfER9AbpfnOh2&search_mode=AdvancedSearch&update_back2search_link_param=yes) |
| **4** | #1 AND #2 AND #3  Indexes=SCI-EXPANDED, SSCI, A&HCI, CPCI-S, CPCI-SSH, BKCI-S, BKCI-SSH, ESCI, CCR-EXPANDED, IC Timespan=1988-2020 | **286** |

1. **Pubmed (n=95) searched and exported to endnote on the 15/12/2020**

| 1 | ("Primary health care" or "primary prevention" or "comprehensive health care" or "Primary care Service delivery" or "Mobile clinic*" or "mobile service" or "ambulatory care facilities" or "mobile health unit" or "outpatient clinic*" or "Community health worker*" or "village health teams" or "Health centre*" or "community clinic*" or "maternal health center*" or "Health facility*" or Hospital*or "family medicine" or "primary care") | 119,708 |
| --- | --- | --- |
| 2 | ("Conflict affected*" or conflict or Humanitarian*or "Civil war" or "Relief work" or "internally Displaced person*" or refugee*) | 10,908 |
| 3 | Africa* or Sub-Saharan Africa or West Africa or East Africa or Central Africa | 276,868 |
| 4 | Burundi or Rwanda or Kenya or South Sudan or Tanzania or Uganda or Cameroon or central Africa republic or Sao Tome and Principe or Djibouti or Eritrea or Ethiopia or Somalia or Comoros or Madagascar or Mauritius or Algeria or Egypt or Libya or Morocco or Sudan or Tunisia or Angola or Lesotho or Malawi or Mozambique or Namibia or south Africa or Swaziland or Zambia or Zimbabwe or Benin or Burkina faso or Ivory Cost or Gambia or Ghana or guinea or guinea Bissau or Liberia or Mali or Mauritania or Niger or Nigeria or sierra leone or western Sahara or Democratic Republic of Congo | 252,722 |
| 5 | 1 AND 2 AND 3 AND 4  ("Primary health care" or "primary prevention" or "comprehensive health care" or "Primary care Service delivery" or "Mobile clinic*" or "mobile service" or "ambulatory care facilities" or "mobile health unit" or "outpatient clinic*" or "Community health worker*" or "village health teams" or "Health centre*" or "community clinic*" or "maternal health center*" or "Health facility*" or Hospital*or "family medicine" or "primary care") AND ("Conflict affected*" or conflict or Humanitarian*or "Civil war" or "Relief work" or "internally Displaced person*" or refugee*) AND ((Burundi or Rwanda or Kenya or South Sudan or Tanzania or Uganda or Cameroon or central Africa republic or Sao Tome and Principe or Djibouti or Eritrea or Ethiopia or Somalia or Comoros or Madagascar or Mauritius or Algeria or Egypt or Libya or Morocco or Sudan or Tunisia or Angola or Lesotho or Malawi or Mozambique or Namibia or south Africa or Swaziland or Zambia or Zimbabwe or Benin or Burkina faso or Ivory Cost or Gambia or Ghana or guinea or guinea Bissau or Liberia or Mali or Mauritania or Niger or Nigeria or sierra leone or western Sahara or Democratic Republic of Congo) OR (Africa* or Sub-Saharan Africa or West Africa or East Africa or Central Africa)) Filters: Clinical Study, Clinical Trial, Comparative Study, Controlled Clinical Trial, Journal Article, Observational Study, Randomized Controlled Trial, Humans, English, from 1988/1/1 - 2020/12/31 | **95** |
