# Supplementary material for: Primary healthcare delivery models in African conflict-affected settings: a systematic review
Source: Confl Health. 2023 Jul 15;17:34. doi: 10.1186/s13031-023-00533-w (PMC10349495; doi:10.1186/s13031-023-00533-w)
Supplement: Supplementary file 2 — Additional file 2. Characteristics of included literature. [file 13031_2023_533_MOESM2_ESM.docx]

**APPENDIX 2**

**Characteristics of included literature**

| **Literature characteristics** | | **No. (%) of studies (n=48)** |
| --- | --- | --- |
| **Year of publication** | |  |
| 1992 - 2005 | Ghebreyesus et al (36); Rutta et al (37) | 2 (4%) |
| 2006 - 2015 | Bolton et al (38); Chamla et al (39); Orach et al (40); Shaikh (41); Colombatti et al (42); Kruk et al (43); Baingana and Mangen (44); Casey et al (45); McGinn et al (46); Zraly et al (47); Kohli et al (48); Casey et al (49); Jordans et al (50); Kersten et al (51); Orach et al (52); Derderian (53); Adam (54); Curry et al (55); Curry et al (56); Gerstl et al (57); Ho et al (58) | 21(44%) |
| 2016 - 2020 | Buesselera and Yugib (59); M Shuaibu et al (60); Casey and Tshipamba (61); Eltayeb et al (62); Izudi et al (63); Murphy et al (64); Ruckstuhl et al (65); Sami et al (66); Murphy et al (67); Kozuki et al (68); Meyer-Weitz et al (69); Odjidja (70); Rull et al (71); Wanzira et al (72); Amsalu et al (73); Bernasconi et al (74); Djerandouba et al (75); Oladeji et al (76); Kumar Das et al (77); Abdullahi et al (78); Altare et al (79); Ataullahjan et al (80); Malembaka et al (81), O'Laughlin et al (82); O'Laughlin et al (83) | 25(52%) |
| **Type of Study** | |  |
| Cross Sectional | Chamla et al (39); Kruk et al (43); Casey et al (45); McGinn et al (46); Casey et al (49); Orach et al (52); Adam (54); Casey and Tshipamba (61); Izudi et al (63); Sami et al (66); Odjidja (70); Wanzira et al (72); Amsalu et al (73); Bernasconi et al (74); Malembaka et al (81) | 15 (31%) |
| Randomized controlled Trail | Bolton et al (38) | 1(2%) |
| Intervention studies | Ghebreyesus et al (36); Shaikh (41); Colombatti et al (42); Baingana and Mangen (44); Zraly et al (47); Derderian (53); Curry et al (55); Curry et al (56); M Shuaibu et al (60); Eltayeb et al (62); Ruckstuhl et al (65); Murphy et al (67); Rull et al (71); Oladeji et al (76); Kumar Das et al (77); Abdullahi et al (78) | 16 (33%) |
| Qualitative methods | Rutta et al (37); Ho et al (58); Buesselera and Yugib (59); Murphy et al (64) | 4(8%) |
| Mixed Methods | Kersten et al (51); Meyer-Weitz et al (69); Djerandouba et al (75); Altare et al (79); Ataullahjan et al (80); Kozuki et al (68) | 6(13%) |
| Economic Evaluation | Orach et al (40) | 1(2%) |
| Pre-Post Study Design | Gerstl et al (57) | 1(2%) |
| Prospective studies | O'Laughlin et al (82); O'Laughlin et al (83) | 2(4%) |
| Case Studies | Jordans et al (50); Kohli et al (48) |  |
| **Phase of Crises** | |  |
| Acute | Kozuki et al 2018; Rull et al 2018 | 2(4%) |
| Protracted | McGinn et al (46); Murphy et al (64); Oladeji et al (76); Orach et al (52); Ruckstuhl et al (65); Sami et al (66); Shaikh (41); Abdullahi et al (78); Adam (54); Altare et al (79); Amsalu et al (73); Ataullahjan et al (80); Buesselera and Yugib (59); Casey et al (45); Casey et al (49); Casey and Tshipamba (61); Chamla et al (39); Djerandouba et al (75); Eltayeb et al (62); Gerstl et al (57); Ho et al (58); Izudi et al (63) ; Jordans et al (50); Kersten et al (51); Kohli et al (48); M Shuaibu et al (60); Curry et al (55, 56); O’Laughlin et al (82, 83); Meyer-Weitz (69); Orach et al (40); Rutta et al (37); Murphy et al (67); Wanzira et al (72) | 36(75%) |
| Post conflict (post emergency) | Baingana and Mangen (44); Bernasconi et al (74); Bolton et al (38); Derderian (53); Ghebreyesus et al (36); Kruk et al (43); Malembaka et al (81); Odjidja (70); Zraly et al (47); Colombatti et al (42) | 10 (21%) |
| **Implementing Institution** | | |
| International Non-Governmental Organisations (N=29) | Adam(54); Abdullahi et al (78); Bernasconi et al(74); Buesselera and Yugib (59); Casey et al (45); Casey and Tshipamba (61); Curry et al(56); Curry et al(55); Derderian (53); Djerandouba et al (75); Gerstl et al (57); Ho et al(58); Jordans et al(50); Kersten et al(51); Kozuki et al(68); Kruk et al (43); McGinn et al (46); Murphy et al(64); O’Laughlin et al(82); Oladeji et al(76); Orach et al(52); Orach et al(40); Ruckstuhl (65); Rull(71); Rutta et al(37) Sami et al (66); Colombatti et al (42); Murphy et al(67), O’Laughlin et al(84) | |
| Government institutions (N=28) | Abdullahi et al (78); Altare et al (79); Amsalu et al(73); Ataullahjan et al (80); Baingana and Mangen (44); Casey et al(49); Casey et al (45); Casey and Tshipamba (61); Chamla et al(39); Curry et al(56); Curry et al(55); Derderian (53); Djerandouba et al (75); Gerstl et al (57); Ghebreyesus et al(36), Ho et al(58); Izudi et al(63); Kruk et al (43); M Shuaibu et al (60); Malembaka et al (81); McGinn et al (46); Meyer-Weitz et al (69); Murphy et al(64); Rutta et al(37); Colombatti et al (42); O’Laughlin et al (84); Kumar Das et al (77); Wanzira et al(72) | |
| National Non-Governmental Organisations (N=12) | Altare et al (79); Baingana and Mangen (44); Bernasconi et al(74); Bolton et al(38); Casey et al(49); Derderian (53); Kruk et al (43); Shaikh (41); Kumar Das et al (77) | |
| United Nations agencies (N=8) | Altare et al (79); Ataullahjan et al (80); Orach et al(52); Orach et al(40); Rutta et al(37); Shaikh (41); Kumar Das et al (77) | |
| Private institution (N=4) | Izudi et al(63); Malembaka (81); Meyer-Weitz et al (69); Rutta et al(37) | |
| Faith Based Organisations (N=3) | Altare et al (79); Derderian (53); Orach et al (40) | |
| Academics/researchers(N=3) | Bolton et al(38); Eltayeb (62); O’Laughlin et al (84) | |
| Community based Organisations (N=2) | Abdullahi et al (78); Orach et al (52) | |
