# Supplementary material for: Primary healthcare delivery models in African conflict-affected settings: a systematic review
Source: Confl Health. 2023 Jul 15;17:34. doi: 10.1186/s13031-023-00533-w (PMC10349495; doi:10.1186/s13031-023-00533-w)
Supplement: Supplementary file 3 — Additional file 3. Systematic review results with man characteristics. [file 13031_2023_533_MOESM3_ESM.docx]

**APPENDIX 3**

**Table of Systematic Review results with main characteristics (selected variables)**

| **Ref. No** | **Authors** | **Title** | **Year of publication** | **Study design** | **Quality rating** | **Study Country** | **Phase of the crises (conflict)** | **Type of models of care** | **Implementing organisation/institution** | **Target population (refugees, IDPs or host communities)** | **list of Services offered** | **Human resources used** |
| --- | --- | --- | --- | --- | --- | --- | --- | --- | --- | --- | --- | --- |
| 35 | Abdullahi et al | Providing TB and HIV outreach services to internally displaced populations in Northeast Nigeria: Results of a controlled intervention study | 2020 | Reports of interventions | High | Nigeria | Protracted conflict | Outreach, Health facilities | Government, CBOs | IDPs, Host communities | Tuberculosis, HIV, Health education, Deworming, Vitamin supplementation | Laboratory Technicians, Doctors, Lay person, Not Specified |
| 36 | Adam | The influence of maternal health education on the place of delivery in conflict settings of Darfur, Sudan | 2015 | Cross sectional | High | Sudan | Protracted conflict | Health facilities, Home visits | INGOs | IDPs | SRH, Immunization, HIV, Acute malnutrition, WASH, Health education | Nurses, Doctors, midwives, medical assistants, Lay person |
| 37 | Altare et al | Health services for women, children and adolescents in conflict affected settings: experience from North and South Kivu, Democratic Republic of Congo | 2020 | Mixed methods | High | Democratic Republic of Congo | Protracted conflict | Health facilities, Community based interventions, Mobile clnics, Outreach | UN agencies, NNGOs, FBOs, Government | IDPs | SRH, Acute malnutrition, ARI, Diarrhea, Malaria, Immunization, WASH, Health education, Immunization | Nurses, CHWs, Doctors |
| 38 | Amsalu et al | Essential newborn care practice at four primary health facilities in conflict affected areas of Bossaso, Somalia: a cross-sectional study | 2019 | Cross sectional | High | Somalia | Protracted conflict | Health facilities | Government | IDPs, Host communities | SRH, Health education | midwives, Nurses |
| 39 | Ataullahjan et al | C'est vraiment compliquÃ©: a case study on the delivery of maternal and child health and nutrition interventions in the conflict-affected regions of Mali | 2020 | Mixed methods | High | Mali | Protracted conflict | Health Facilities | UN agencies, INGOs, NNGOs, Government | IDPs | SRH, Acute malnutrition | Not Specified |
| 40 | Baingana and Mangen | Scaling up of mental health and trauma support among war affected communities in northern Uganda: lessons learned | 2011 | Reports of interventions | Low | Uganda | Post-conflict | Health facilities, Community based interventions, Outreach | NNGOs, Government | IDPs | Mental health | Nurses, Lay person, social workers, Clinical officer |
| 41 | Bernasconi et al | Results from one-year use of an electronic Clinical Decision Support System in a post-conflict context: An implementation research | 2019 | Cross sectional | High | Nigeria | Post-conflict | Health facilities | INGOs, NNGOs | conflict affected communities | ARI, Diarrhea, Malaria, Acute malnutrition, Immunization, Survellaince, Deworming, Vitamin supplementation | Laboratory Technicians, midwives, Nurses, Clinical officers |
| 42 | Buesselera and Yugib | Childbirth in South Sudan: preferences, practice and perceptions in the Kapoetas | 2016 | Qualitative study | Low | South Sudan | Protracted conflict | Health facilities | INGOs | IDPs | SRH | Nurses, Doctors |
| 43 | Casey et al | Availability of long-acting and permanent family-planning methods leads to increase in use in conflict-affected northern Uganda: evidence from cross-sectional baseline and endline cluster surveys | 2013 | Cross sectional | High | Uganda | Protracted conflict | Mobile clnics, Community based interventions, Health facilities | NNGOs, Government | IDPs, conflict affected communities | SRH, Health education | Doctors, midwives, Nurses, CHWs, village health teams |
| 44 | Casey and Tshipamba | Contraceptive availability leads to increase in use in conflict-affected Democratic Republic of the Congo: evidence from cross-sectional cluster surveys, facility assessments and service statistics | 2017 | Cross sectional | High | Democratic Republic of Congo | Protracted conflict | Health facilities | INGOs, Government | IDPs, conflict affected communities | SRH, Health education | Not Specified |
| 45 | Chamla et al | Geographical information system and access to HIV testing, treatment and prevention of mother-to-child transmission in conflict affected Northern Uganda | 2007 | Cross sectional | High | Uganda | Protracted conflict | Health facilities | Government | IDPs, Host communities | HIV | Clinical officers |
| 46 | Curry et al | Delivering high-quality family planning services in crisis-affected settings II: results | 2015 | Reports of interventions | medium | Chad, DRC, Dibouti, Mali | Protracted conflict, Host country | Health facilities | INGOs, Government | IDPs, Refugees, Host communities | SRH | Nurses, Doctors, CHWs, Laboratory Technicians |
| 47 | Curry et al | Delivering high-quality family planning services in crisis-affected settings I: program implementation | 2015 | Reports of interventions | High | Chad, DRC, Dibouti, Mali | Protracted conflict, Host country | Health facilities | INGOs, Government | IDPs, Host communities, Refugees | SRH, Health education | Nurses, Laboratory Technicians, CHWs, Doctors |
| 48 | Derderian | Changing tracks as situations change: humanitarian and health response along the Liberia-Cote d'Ivoire border | 2014 | Reports of interventions | Low | Liberia, Ivory Coast | Post-conflict | Mobile clnics, Health facilities | INGOs, Government, NNGOs, FBOs | Refugees, IDPs |  | Nurses, Doctors |
| 49 | Eltayeb et al | Bridging the gap in mental health and psychosocial services in low resource settings: a case study in Sudan | 2017 | Reports of interventions | medium | Sudan | Protracted conflict | Community based interventions, Health facilities | Researchers/acadamic institutions | IDPs | Mental health | Doctors, CHWs, Pyschologist |
| 50 | Ho et al | Effects of a community scorecard on improving the local health system in eastern Democratic Republic of Congo: qualitative evidence using the most significant change technique | 2015 | Qualitative study | High | Democratic Republic of Congo | Protracted conflict | Health facilities, Community based interventions | INGOs, Government | conflict affected communities |  | Nurses |
| 51 | Izudi et al | Early postnatal care use by postpartum mothers in Mundri East County, South Sudan | 2017 | Cross sectional | High | South Sudan | Protracted conflict | Health facilities, Community based interventions | Government, Private institution | conflict affected communities | SRH, Immunization | CHWs, Doctors, Nurses, midwives |
| 52 | Kersten et al | Too complicated for the field? Measuring quality of care in humanitarian aid settings | 2013 | Mixed methods | medium | South Sudan | Protracted conflict | Mobile clnics, Health facilities, Outreach | INGOs | conflict affected communities | Acute malnutrition, Injuries and Trauma, Immunization, SRH, Malaria, Survellaince, Tuberculosis, ARI, Diarrhea | Not Specified |
|  |  |  |  |  |  |  |  |  |  |  |  |  |
| 53 | Kozuki et al | The resilience of integrated community case management in acute emergency: A case study from south sudan | 2018 | Mixed methods | High | South Sudan | Acute Conflict | Community based interventions, Health facilities | INGOs | IDPs, Host communities | ARI, Diarrhea, Malaria, Health education | CHWs |
| 54 | Kruk et al | Availability of essential health services in post-conflict Liberia | 2009 | Cross sectional | High | Liberia | Post-conflict | Health centers | Government, NNGOs, INGOs | conflict affected communities | HIV, SRH, Diarrhea, ARI, Malaria, Mental health, Immunization | Nurses |
| 55 | Malembaka et al | Are people most in need utilizing health facilities in post-conflict settings? A cross-sectional study from south Kivu, eastern DR Congo | 2020 | Cross sectional | High | Democratic Republic of Congo | Post-conflict | Health centers, Community based interventions | Government, Private institution | conflict affected communities | Acute malnutrition, NCDs | Nurses, CHWs |
| 56 | McGinn et al | Family planning in conflict: results of cross-sectional baseline surveys in three African countries | 2011 | Cross sectional | medium | Sudan, Uganda, Democratic Republic of Congo | Protracted conflict | Health centers, Community based interventions | INGOs, Government | conflict affected communities | SRH, Health education | Not Specified |
| 57 | Meyer-Weitz et al | Healthcare service delivery to refugee children from the Democratic Republic of Congo living in Durban, South Africa: a caregivers' perspective | 2018 | Mixed methods | High | South Africa | Host country | Health centers | Government, Private institution | Refugees |  | Doctors, Nurses |
| 58 | Murphy et al | Diabetes care in a complex humanitarian emergency setting: a qualitative evaluation | 2017 | Qualitative study | High | Democratic Republic of Congo | Protracted conflict | Health centers | INGOs, Government | IDPs | NCDs | Nurses, Doctors, medical assistants, Nutritionist, Nurses, Doctors, medical assistants, Nutritionist |
| 59 | O'Laughlin et al | The cascade of HIV care among refugees and nationals in Nakivale Refugee Settlement in Uganda | 2017 | Prospective studies | High | Uganda | Host country | Health centers | INGOs | Refugees, Host communities | HIV | Not Specified |
| 60 | Oladeji et al | Integrating immunisation services into nutrition sites to improve immunisation status of internally displaced persons' children living in bentiu protection of civilian site, south sudan | 2019 | Reports of interventions | High | South Sudan | Protracted conflict | Health facilities, Outreach, Community based interventions | INGOs | IDPs | Immunization, nutrition, Health education | CHWs |
| 61 | Orach etal, | Accessibility and availability of health care services to internally displaced persons, in Kitgum and Pader districts, northern Uganda | 2013 | Cross sectional | medium | Uganda | Protracted conflict | Health centers | UN agencies, INGOs, CBOs, UN agencies | IDPs | Health education | nurse assistant |
| 62 | Orach et al | Costs and coverage of reproductive health interventions in three rural refugee-affected districts, Uganda | 2007 | Economic evaluation study | medium | Uganda | Host country | Health centers | UN agencies, INGOs, FBOs, Government | Refugees, Host communities | SRH | Nurses, Doctors |
| 63 | Rull et al | The new WHO decision-making framework on vaccine use in acute humanitarian emergencies: MSF experience in Minkaman, South Sudan | 2018 | Reports of interventions | High | South Sudan | Acute Conflict | Health facilities, Outreach | INGOs | IDPs | Immunization, Diarrhea, WASH, Survellaince, Acute malnutrition, SRH | CHWs |
| 64 | Rutta et al | Refugee perceptions of the quality of healthcare: findings from a participatory assessment in Ngara, Tanzania | 2005 | Qualitative study | High | Tanzania | Host country | Community based interventions, Home visits, Health facilities | INGOs, Government, Private institution, UN agencies | Refugees | Acute malnutrition, Health education, HIV, Mental health, SRH, Immunization | CHWs, TBAs |
| 65 | Sami et al | State of newborn care in South Sudan's displacement camps: a descriptive study of facility-based deliveries | 2017 | Cross sectional | High | South Sudan | Protracted conflict | Health facilities | INGOs | IDPs, Refugees | SRH, Health education | midwives, CHWs, TBAs |
| 66 | Colombatti et al | A short-term intervention for the treatment of severe malnutrition in a post-conflict country: results of a survey in Guinea Bissau | 2008 | Reports of interventions | High | Guinea Bissau | Post-conflict | Health centers | Government, INGOs | conflict affected communities | Acute malnutrition, Deworming, Malaria, ARI | Nurses |
| 67 | Murphy et al | Management of acute malnutrition in infants less than six months in a South Sudanese refugee population in Ethiopia | 2017 | Reports of interventions | medium | Ethiopia | Host country | Community based interventions, Health centers, Outreach | INGOs, UN agencies | Refugees | Acute malnutrition, Health education | CHWs |
| 68 | Sanjay et al | Nutritional response in north-eastern Nigeria: Approaches to increase service availability in Borno and Yobe States | 2019 | Reports of interventions | medium | Nigeria | Protracted conflict | Health centers, Outreach, Mobile clnics, PHC clinics, Community based interventions | UN agencies, Government, NNGOs | IDPs | Acute malnutrition, Health education | CHWs |
| 69 | Wanzira et al | Quality of care for children with acute malnutrition at health center level in Uganda: a cross sectional study in West Nile region during the refugee crisis | 2018 | Cross sectional | High | Uganda | Host country | Community based interventions, Health facilities | Government | Refugees | Acute malnutrition, HIV, Tuberculosis | Nurses, midwives, Clinical officers, Nurse assistant |
| 70 | Gerstl et al | High adherence to malaria treatment: promising results of an adherence study in South Kivu, Democratic Republic of the Congo | 2015 | Pre-post intervention | High | Democratic Republic of Congo | Protracted conflict | Health facilities | INGOs, Government | conflict affected communities | Malaria | Not Specified |
| 71 | Casey et al | Care-seeking behavior by survivors of sexual assault in the Democratic Republic of the Congo | 2011 | Cross sectional | Low | Democratic Republic of Congo | Protracted conflict | Health facilities | Government, INGOs | conflict affected communities | SRH | Not Specified |
| 72 | O'Laughlin et al | Feasibility and acceptability of home-based HIV testing among refugees: a pilot study in Nakivale refugee settlement in southwestern Uganda | 2018 | non-RCT study | High | Uganda | Host country | Home visits, Health centers | INGOs, Government, Researchers/acadamic institutions | Refugees, Host communities | HIV |  |
| 73 | Djerandouba et al | Evaluation of the effectiveness of community health workers in the fight against malaria in the Central African Republic (2012-2017) | 2019 | Mixed methods | High | Central African Republic | Protracted conflict | Community based interventions | INGOs, Government | conflict affected communities | Malaria, Health education, Deworming, Diarrhea | CHWs |
| 74 | Ghebreyesus et al | Community participation in malaria control in Tigray region Ethiopia | 1996 | Reports of interventions | High | Ethiopia | Post-conflict | Community based interventions | Government | conflict affected communities | Malaria, Health education | CHWs |
| 75 | Jordans et al | Implementation of a mental health care package for children in areas of armed conflict: a case study from Burundi, Indonesia, Nepal, Sri Lanka, and Sudan | 2013 | Case studies | medium | Sudan, Burundi | Protracted conflict | Community based interventions | INGOs | conflict affected communities | Mental health, Health education | Pyschologist, counsellor |
| 76 | Kohli et al | A Congolese community-based health program for survivors of sexual violence | 2012 | Case studies | High | Democratic Republic of Congo | Protracted conflict | Mobile clnics, Community based interventions | NNGOs | conflict affected communities | SRH, Health education, HIV, Mental health, Malaria | Doctors, Nurses |
| 77 | Ruckstuhl et al | Malaria case management by community health workers in the Central African Republic from 2009-2014: overcoming challenges of access and instability due to conflict | 2017 | Reports of interventions | High | Central African Republic | Protracted conflict | Community based interventions | INGOs | IDPs, Host communities | Malaria, Health education, Acute malnutrition, Deworming, Survellaince, Vitamin supplementation | CHWs |
| 78 | Zraly et al | Primary mental health care for survivors of collective sexual violence in Rwanda | 2011 | Reports of interventions | High | Rwanda | Post-conflict | Community based interventions | NNGOs | conflict affected communities | HIV, Tuberculosis, Mental health | CHWs, Lay person |
| 79 | Bolton et al | Interventions for depression symptoms among adolescent survivors of war and displacement in northern Uganda - A randomized controlled trial | 2007 | Randomized Control trails | High | Uganda | Post-conflict | Community based interventions | Researchers/acadamic institutions, NNGOs | IDPs | Mental health | Not Specified |
| 80 | M Shuaibu et al | Mass immunization with inactivated polio vaccine in conflict zones - Experience from Borno and Yobe States, North-Eastern Nigeria | 2016 | Reports of interventions | High | Nigeria | Protracted conflict | Mobile clnics, Outreach | Government | IDPs, Host communities | Immunization, Survellaince, Malaria, ARI, Diarrhea | midwives, Doctors, medical assistants |
| 81 | Odjidja | Control of infectious disease during pregnancy among pastoralists in South Sudan: A case for investment into mobile clinics | 2018 | Cross sectional | Low | South Sudan | Post-conflict | Mobile clnics |  | conflict affected communities | Malaria, Immunization, Tuberculosis, SRH, HIV | Nurses, medical assistants |
| 82 | Shaikh | Nurses' use of global information systems for provision of outreach reproductive health services to internally displaced persons | 2008 | Reports of interventions | medium | Somalia | Protracted conflict | Mobile clnics | NNGOs, UN agencies | IDPs | SRH, Health education | Nurses, midwives, Nurse assistant |
